# Quantifying water distribution between starch and protein in doughs and gels from mildly refined faba bean fractions

**DOI:** 10.1016/j.crfs.2022.03.013

**Published:** 2022-04-14

**Authors:** Jan M. Bühler, Atze Jan van der Goot, Marieke E. Bruins

**Affiliations:** a Food & Biobased Research, Wageningen University & Research, Bornse Weilanden 9, 6708 WG, Wageningen, the Netherlands; b Food Process Engineering, Agrotechnology and Food Sciences Group, Wageningen University & Research, Bornse Weilanden 9, 6708 WG, Wageningen, the Netherlands

**Keywords:** Starch gelatinization, Protein denaturation, Faba bean concentrate, Mild processing, Moisture distribution, Starch swelling, DSC, Differential Scanning Calorimetry, FM, Faba bean meal, PF, Protein fraction, SF, Starch fraction, TD-NMR, Time Domain Nuclear Magnetic Resonance

## Abstract

The development of novel and sustainable food products, such as cheese- and meat analogues, requires a better understanding of the use of less refined ingredients. We investigated the distribution of water between the protein and starch phase of doughs and heat-induced gels made from air-classified faba bean fractions by developing a method suited for investigation of such multi-component ingredients. The moisture contents of the protein and starch phases in the dough were determined using a method based on partial sorption isotherms of mixed doughs of protein- and starch-rich fractions at high water activity. Water content of the protein phase is higher than that of the starch phase in dough, showing that protein takes up more water than starch at room temperature. Also, the moisture content of the protein phase in the gels was calculated using a model based on the denaturation temperature of legumin. From the experiments and the modelling, it became evident that the moisture content of the protein phase in the gel is lower than the moisture content of the protein phase in the dough, showing the importance of considering moisture migration from the protein to the starch during heating.

## Highlights

• Water distribution among phases in doughs and gels is measured “in-situ”.
• Addition of starch increases water content of protein phase in doughs.
• Water migrates from protein to starch after initial starch gelatinization.

## Graphical abstract

**Figure:**
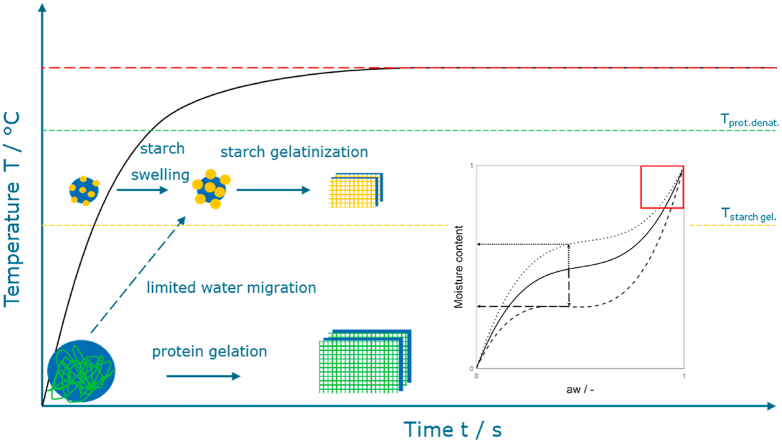

## 1 Introduction

To make attractive new food products, we have to understand how sensory properties of the product depend on components and their interactions with water. This understanding is especially important in soft-solid products like cheeses and meat but even more so for the plant-based alternatives of those products. Thus far, the products are mainly based on soy, pea or wheat, but interest in the use of other raw materials is increasing. Faba beans are considered as one of the promising new crops to provide healthy plant proteins (Multari et al., 2015). It is therefore taken as an exemplary material of a starch-containing crop in this study.

Meat and cheese analog products are characterized by having a dry matter content of around 20–40% in the water phase, which gives high water activity. Besides, multiple aqueous phases can be present in those products. The water content in each phase of such materials has a large influence on properties such as gelling behavior and thereby on the properties of the final product. Thus far, research on the influence of starch on protein gelation and vice-versa, as well as research on the influence of water content on product properties of soft-solid products, is often limited to the use of pure components, meaning isolates (Dekkers et al., 2016; Aguilera and Baffico, 1997; Eliasson, 1983; Walsh et al., 2008; Zhang et al., 2016; Fernández-Gutiérrez et al., 2004). The use of less refined ingredients would improve the sustainability and cost efficiency of these products, for example meat analogues (Berghout et al., 2015; Schutyser et al., 2015; van der Goot et al., 2016). In order to use less refined ingredients for making food products, the interactions of the components present in less refined ingredients needs to be understood. This can only be achieved by performing experiments directly on the less refined ingredients instead of mixtures of highly purified ingredients, where protein and starch structure and therefore functionality have been modified during the purification process applied to make the ingredients (Bühler et al., 2021).

Starch gelatinization is a clear example of the effect of water content on food properties. Although the initial gelatinization of starch occurs at a gelatinization temperature independent of the moisture content, the magnitude of gelatinization at said temperature decreases with the water content (Donovan, 1979; Eliasson, 1980; Tananuwong and Reid, 2004). In case of incomplete gelatinization of starch, a second transition occurs at higher temperatures. This transition is the melting of starch crystals, and the melting temperature depends on the available water (Tananuwong and Reid, 2004). Additives that modify the water activity (and therefore the water availability) also influence the gelatinization parameters of starch (Wootton and Bamunuarachchi, 1980). Similarly, the gelling parameters of protein are influenced by the available water as well. Barker (1933) found a linear correlation between the decrease of the denaturation temperature of egg albumin and the moisture content. Since then, similar relations have been established for many proteins, including vicilin and legumin in faba bean (Arntfield et al., 1985). As the gelling properties of protein and the gelatinization properties of starch both depend on the available moisture, determining this available moisture is essential to understand the behavior of food products that contain both starch and proteins. In a mixed dough or gel, starch and protein are often seen as individual phases that do not mix on a molecular level due to thermodynamic incompatibility (Grinberg and Tolstoguzov, 1997; Tolstoguzov, 1997, 2006; Bot and De Bruijne, 2003). Due to different affinities for water, the available moisture in these two phases will differ from the overall moisture content, which is an average value of the different moisture contents in the different phases. Often this water distribution is not measured, but obtained via fitting the water distribution between phases in a biopolymer blend or assumed from the overall moisture content when describing for example rheological properties by applying the polymer blending law to starch-protein mixtures or mixed protein blends (Aguilera and Baffico, 1997; Shrinivas and Kasapis, 2009; Fitzsimons et al., 2008; Clark et al., 1983). In addition, Dekkers et al. (2016) developed a method based on TD-NMR to determine water distribution in mixed protein gels. However, it requires the availability of and information about the pure components used to make the mixed gels. For many food products, it is not possible to obtain the components in pure form, especially in the same physical state as in the product. For example, a purification process might involve a heating step, which changes properties of starches and proteins, which can change the distribution of water among them. The use of model mixtures of protein isolates and starch isolates will therefore not accurately reflect the behavior of real ingredients (Bühler et al., 2021).

The aim of this research is to determine how starch and protein content influence the water distribution in unheated doughs and heat-induced gels that are prepared from mixtures of mildly refined ingredients. For this, we developed new methods for the determination of the water distribution in doughs at room temperature and for gels at elevated temperatures that do not depend on pure components that are otherwise difficult to obtain in the same, native form in which they are present in the mildly refined fractions. The method for the doughs combines fundamental principles of sorption isotherms with mass balances. Modelling is used to calculate the contribution of each pure component, which is then transferred into a sorption isotherm for the pure component at high water activity (*a*_*w*_ > 0.96). This high *a*_*w*_ is relevant when considering products like meat and cheese analogues that have a moisture content between 40 and 80% Kyriakopoulou et al. (2019); Cornet et al. (2021); Grasso et al. (2021). However, this range of water activity is rarely investigated, causing a limitation of available data to compare to for both faba bean (Alpizar-Reyes et al., 2018; Menkov, 2000) and starch in general (Al-Muhtaseb et al., 2004). The method to determine the distribution of moisture among the phases of gels at elevated temperatures is based on the denaturation temperature of the protein, measured with DSC. These methods enable us to understand the changes in water distribution due to a thermal treatment under the relevant conditions. These changes might have implications for research on the properties of such mixed doughs and gels that depend on the water distribution, for example determining and predicting sensory attributes of protein/starch gel products (meat analogues, cheese analogues, sauces, dairy analogues).

Faba bean Meal (FM) is taken as an exemplary material. FM can be dry fractionated, delivering protein and starch enriched fractions with native properties. This ensures that neither protein nor starch are denatured or otherwise influenced by the fractionation method, therefore retaining their native functionality. Furthermore, FM contains enough starch to allow a starch content range in mixed doughs of 0.09–0.66 g g^−1^ (db.) while ensuring that protein content is high enough to allow the detection of its denaturation.

## 2 Materials & methods

Faba bean Meal HOMECRAFT®Pulse 3101 (FM) was supplied by Ingredion (Hamburg, Germany). FM had a protein content of 29% (N-conversion factor = 6.25). FM was air classified using a Hosokawa Multi Mill (Alpine, Augsburg, Germany). The classifier wheel speed was set to 7000 rpm and the air flow to 70 m^3^h^−1^. The composition of the protein-rich fine fraction (Protein Fraction - PF) and the starch-rich coarse fraction (Starch Fraction - SF) is shown in Table 1.

**Table 1:** Composition of PF and SF in mass %. Starch content was determined using MegaZyme Starch Kit, protein content was determined using DUMAS (N-conversion factor = 6.25). Moisture content was determined using oven drying at 105 °C until the weight remained constant.

	Starch content / %	Protein content / %	Moisture content / %
PF	8.5	58.8	8.2
SF	66.3	15.5	6.79

PF and SF were mixed at different ratios to vary starch content (9–70% d.b.). Starch content will be expressed in g g^−1^ as w/w % on d.b., unless specified otherwise. Mixtures were combined with distilled water to create doughs with different moisture content (36–66% wet basis), for which the moisture content of the powders was taken into account. Moisture content or dry matter content are expressed in g g^−1^ on wet basis, unless specified otherwise. Doughs were stored in vacuum-sealed bags at 4 °C for 48 h to allow water distribution to reach equilibrium.

### 2.1 Water activity

Water activity (*a*_*w*_) of the doughs was measured using an Aqualab TDL water activity meter (METER Group, Pullman, USA). Approximately 3 g of sample were used for each measurement. The measuring temperature was set to 25 °C with a deviation of 0.05 °C, ensuring constant measuring conditions.

### 2.2 Moisture content

Moisture content (mc) of all doughs was determined by oven drying. Dough samples were dried in aluminum cups at 105 °C for 24 h. Moisture content was calculated according to Equation (1):

(1) $$mc=\frac{m_{\mathrm{wet}}-m_{\mathrm{dry}}}{m_{\mathrm{wet}}}$$

Where *m*_*wet*_ is the weight of the wet sample and *m*_*dry*_ is the weight of the dry sample. Moisture contents are always expressed on a wet basis (wb.), unless specified otherwise.

### 2.3 Differential Scanning Calorimetry (DSC)

Differential Scanning Calorimetry (DSC) was used to determine the denaturation temperature of the proteins and the degree of starch pasting. Dough samples were degassed in an ultrasonic water bath for 15 min 60 mg was transferred to High Volume Pans (100 μl, TA Instruments, New Castle, USA). The pans were placed in the DSC (DSC-250, TA Instruments, New Castle, USA) where they were first equilibrated at 20 °C until the temperature was constant. Samples were then heated with a ramp of 5 °C min^−1^ to 160 °C. After cooling, the cycle was repeated. TRIOS software was used to analyze the obtained thermograms and to identify the peak temperatures of the protein denaturation and the enthalpy change of the initial peak of starch pasting (*G* peak). Δ*H* was obtained by adjusting for the amount of starch present in the sample using

(2) $$\Delta H_{\mathrm{Starch}}=\frac{\Delta H}{\left(1-mc\right)*sc}$$

where Δ*H* is the enthalpy change per overall sample mass in J g^−1^, *mc* is the overall moisture content in g g^−1^ (wb.) and *sc* is the starch content in g g^−1^ (db.).

### 2.4 Modelling and statistics

All measurements are shown as individual data points in the graphs. Multiple Linear Regression was used to fit the data in R (Version 3.6.1). For the dough, the model Equations (3), (4), (5), (6)) were used:

(3) mc=a*sc+d

(4) $$mc=a*a_{w}+d$$

(5) $$mc=a*a_{w}+b*sc+d$$

(6) $$mc=a*a_{w}+b*sc+c*a_{w}*sc+d$$

where *mc* is the overall moisture content of the sample in g g^−1^ (wb.), *a*_*w*_ is the water activity, *sc* is the starch content in g g^−1^ (db.) and a, b, c and d are model parameters. For the model of the gel, the same method was used with the model Equations (7)–(10).

(7) $$T_{d}=e*sc+h$$

(8) $$T_{d}=e*mc+h$$

(9) $$T_{d}=e*mc+f*sc+h$$

(10) $$T_{d}=e*mc+f*sc+g*mc*sc+h$$

where *T*_*d*_ is the denaturation temperature of legumin in °C, *mc* is the overall moisture content of the sample in g g^−1^ (wb.), *sc* is the starch content in g g^−1^ (db.) and e, f, g and h are model parameters. The residuals of each fit were checked for correlation in R. The summary tables of each model can be found in the Supplementary information. The best fit was chosen based on the value of $R_{\mathrm{adj}}^{2}$ and the significance of the independent variables (p-value).

## 3 Theory

**Fig. 1:**
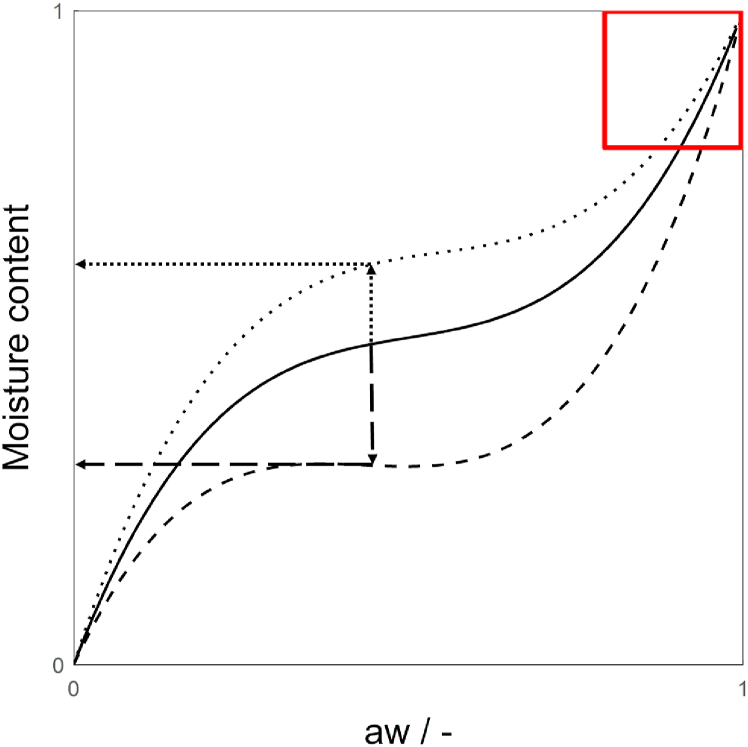
Sketch of sorption isotherms of protein (dotted line), starch (dashed line) and a mixture of the two (solid line), adapted from Barbosa-Cánovas et al. (2007). Arrows indicate the method to obtain the moisture content of the individual phases in the mixture. The red square indicates the area of the curve that is investigated in this study. (For interpretation of the references to colour in this figure legend, the reader is referred to the Web version of this article.)

Starting point of the method to determine the distribution of moisture among phases of a starch-protein blend at room temperature (called “dough”) presented in this paper is the fact that the water activity of such a blend is determined by both the overall moisture content and the composition of the dough (Barbosa-Cánovas et al., 2007) (Fig. 1). The assumption of full phase separation between starch and protein makes it possible to use the sorption isotherm of the blend to obtain the isotherms of the individual phases (Labuza and Hyman, 1987). The moisture content of the individual phases in the dough can then be read from the sorption isotherm of the individual phases at the measured *a*_*w*_ of the dough. At moisture contents higher than 40%, the water activity of the product is between 0.9 and 1 and their relation can be approximated well by a straight line. The shape of the sorption isotherm of starch and protein only deviates from a linear relation at *a*_*w*_ < 0.95 (Lomauro et al., 1985; Xu et al., 2019). This simple method to determine the water available to starch and protein can however only be carried out when the mixture is in equilibrium. Therefore, it is not suitable at high temperatures due to evaporation and reactions taking place (*e.g.* gelation). To determine the distribution of moisture at higher temperatures, a method based on the denaturation temperature of the protein is proposed. As the denaturation temperature of protein depends on the moisture content of the protein phase, the first can be used to determine the latter if the relation is known. We model the relation by extrapolating the measured denaturation temperature of protein of the mixed gels to a protein phase containing no starch, yielding the moisture content of the protein phase in the mixed, heat-induced gel.

Next to the denaturation temperature of protein, the gelatinization of starch can also be monitored. If sufficient water is available, starch granules fully gelatinize at the initial gelatinization temperature (Biliaderis et al., 1980; Haase and Shi, 1991), resulting in the peak of gelatinization (*G* peak) (Tananuwong and Reid, 2004) in a DSC thermogram. Under low moisture conditions, the amorphous regions of the starch granule are not fully hydrated and hence the crystalline regions initially stay partly intact. The crystalline regions of the starch granule then melt at a higher temperature, also resulting in a peak in the DSC thermogram (*M1* peak: high temperature, low moisture melting peak) (Donovan, 1979; Hoseney et al., 1986). The position of this peak depends on the amount of water that can be taken up by the granules during the initial gelatinization. When no water is available, only the *M1* peak is present (Biliaderis et al., 1980). According to Tananuwong and Reid (2004), the sum of the enthalpy change of the *G* and *M1* peak is independent of the overall moisture content. At high moisture contents, the peak temperature of the *M1* peak decreases in a magnitude that it overlaps the *G* peak. Here, this fact is used to take the enthalpy change of the *G* peak of the starch phase (Δ*H*) as a measure of the moisture content of starch in the dough.

## 4 Results & discussion

### 4.1 Moisture distribution in starch-protein doughs at room temperature

In this study, protein-rich fractions (PF) and starch-rich fractions (SF) were mixed to obtain doughs that vary in protein and starch content. Besides, water addition was used to change the water contents. Since PF and SF used in this study were obtained via dry fractionation, it is assumed that starch granules will remain intact (Vogelsang-O’Dwyer et al., 2020). This prevents mixing with the other components on a molecular level and starch can therefore be regarded as a separate phase. The other phase consists of proteins and other components such a fibres and sugars. Also these components will bear native properties, due to the lack of heating in dry fractionation. Despite the presence of the other components, we will describe the latter phase as protein phase, and study how the water will distribute between the starch and this so-called protein phase (Eliasson, 1983).

**Fig. 2:**
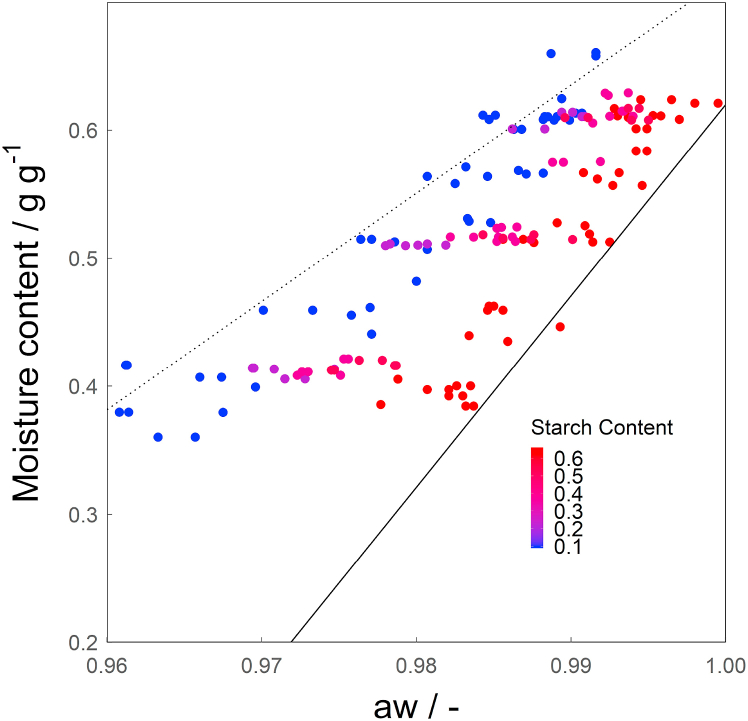
Partial sorption isotherms showing the influence of starch content of the correlation between overall moisture content and water activity. The dotted line represents the protein phase (Equation (12)), the solid line the starch phase (Equation (13)).

The overall moisture content (*mc*) and water activity (*a*_*w*_) of 155 dough samples with different starch and protein contents and added amounts of water were measured (Fig. 2) at 25 °C. The data obtained represents the section of the sorption isotherm where the shape of the curve becomes asymptotic (Baucour and Daudin, 2000), indicated by the red square in Fig. 1. The partial sorption isotherm therefore becomes linear at these high moisture contents. Therefore, the data in Fig. 2 shows linear relations for each starch concentration linking water activity to moisture content. The slopes differ depending on the starch content, leading to a larger difference in *a*_*w*_ at the lower moisture contents than at the highest moisture content.

The potential influence of the starch content on the correlation between the moisture content and the water activity can be incorporated in a model via Equations (3)–(6). It is important to note that the dry based protein content (*pc*) and the dry based starch content (*sc*) of the samples can be expressed as *sc* + *pc* = 1. This means that protein content was not varied independently of starch content and is therefore not used as an independent variable. Equation (6) was found to give the best fit based on the highest $R_{\mathrm{adj}}^{2}$, while the p-value was below 0.001 for all variables used, showing that they are significant. Summary tables of all fitted models can be found in the Supplementary information (Tables S1–S4). Fitting resulted in the following equation describing the relation between water activity, starch content and moisture content in starch-protein doughs:

(11) $$mc=8.46*a_{w}-6.58*sc+6.48*a_{w}*sc-7.74$$

where *mc* is the overall moisture content of the sample in g g^−1^ (wb.), aw is the water activity, and *sc* is the starch content in g g^−1^ (db.). This optimized fit resulted in $R_{\mathrm{adj}}^{2}=0.89$. The investigated range is limited to the moisture content relevant for protein/starch gel products such as meat- and cheese analogues.

The moisture content of the protein phase as well as the starch phase in a sample at a given water activity can be calculated from Equation (11) by setting the starch content or respectively the protein content to zero. This yields the moisture content of the protein phase in the dough (*mc*_*p*_(*dough*), Equation (12)) and the moisture content of the starch phase in the dough (*mc*_*s*_(*dough*), Equation (13)):

(12) $$mc_{p}\left(dough\right)=8.46*a_{w}-7.74$$

(13) $$mc_{s}\left(dough\right)=14.94*a_{w}-14.32$$

where *mc*_*i*_(*dough*) is the moisture content of the protein or starch phase in the dough in g g^−1^ (wb.) and *a*_*w*_ is the water activity (−) of the sample. These equations are given as lines in Fig. 2. The linear relation from Equation (11) is supported by the fact that the shape of the sorption isotherm of starch and protein only deviates from a linear relation at lower *a*_*w*_, according to literature: In a study on several raw legume flours (chickpea, lentil and yellow pea), sorption isotherms were measured, for all of which this deviation only occurred at *a*_*w*_ < 0.8 and *mc* < 0.13 g g^−1^ (wb.) (Xu et al., 2019). For corn starch the relation seems to be linear at *a*_*w*_ > 0.9 and *mc* > 0.15 g g^−1^ (wb.) at 30 °C (Peng et al., 2007). All samples used in the further analysis of this paper had an *a*_*w*_ of over 0.97.

**Fig. 3:**
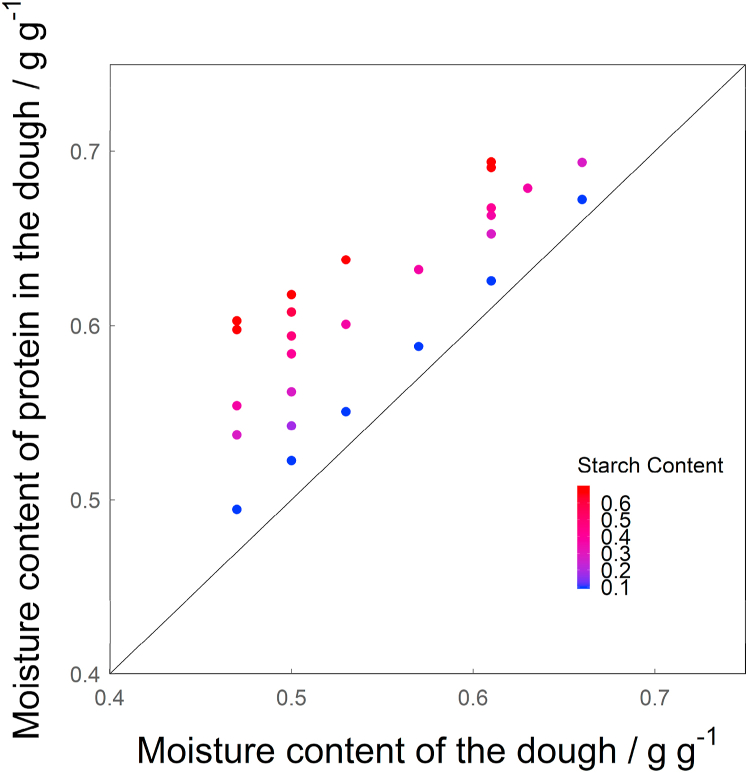
Modelled moisture content in the protein phase in the dough (*mc*_*p*_(*dough*)) over the moisture content of the dough. The solid line represents where the moisture content of the dough and the moisture content of the protein phase in the dough would be the same.

In Fig. 3, the calculated moisture content in the protein phase at room temperature is shown over the overall moisture content for all used starch contents (db.). The moisture content of the protein phase in the dough increases with starch content, depending on the overall moisture content. At lower moisture contents (0.47 g g^−1^), an increase of the starch content from 0.09 to 0.7 g g^−1^ (d.b.) leads to an increase in moisture content of the protein phase of 0.11 g g^−1^ (22%). The same increase in starch content at higher overall moisture content (0.61) only causes an increase of 0.07 g g^−1^ (9%). As the overall moisture content increases and the *a*_*w*_ approaches 1, the sample approaches a regime of excess water, where a change in starch content will not have an influence on the moisture content in protein, as both the moisture content in starch and protein will be equal to the overall moisture content.

### 4.2 Moisture distribution in starch-protein mixtures at increasing temperature

#### 4.2.1 DSC thermograms of starch/protein blends

**Fig. 4:**
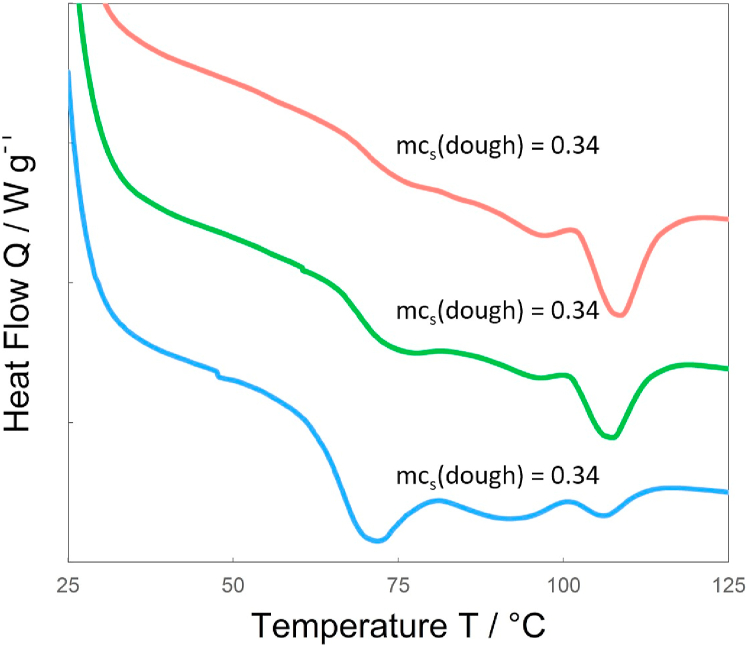
Exemplary DSC thermograms of three samples with (red) 0.28 g g^−1^ starch content (d.b.), 0.51 g g^−1^ moisture content resulting in a *mc*_*s*_(*dough*) = 0.34 g g^−1^, with (green) 0.37 g g^−1^ starch content (d.b.), 0.53 g g^−1^ moisture content resulting in a *mc*_*s*_(*dough*) = 0.41 g g^−1^, and with (blue) 0.66 g g^−1^ starch content (d.b.), 0.61 g g^−1^ moisture content resulting in a *mc*_*s*_(*dough*) = 0.57 g g^−1^. Curves are offset in y-direction to display all three graphs in one figure without overlapping. Tick-marks on y-axis represent steps of 0.02 W g-1. All curves shown are in the range of −0.28 to −0.20 W g-1. Please note that the Heat Flow Q shown here is only normalized for the overall mass, not the mass of starch in the sample. (For interpretation of the references to colour in this figure legend, the reader is referred to the Web version of this article.)

The starch gelatinization as well as the protein denaturation were analyzed using DSC. From the thermograms of the DSC (Fig. 4) we can gain information about changes that occur, such as the peak temperature (T) and the enthalpy change (Δ*H*) associated with the reaction. Fig. 4 shows three exemplary thermograms, one with a low *mc*_*s*_(*dough*) (red), one with an intermediate *mc*_*s*_(*dough*) (green) and one with a high *mc*_*s*_(*dough*) (blue). There are three peaks visible in all three thermograms. The first peak is the initial gelatinization of starch, also called the *G* peak (Tananuwong and Reid, 2004), which occurs when starch is heated above the pasting temperature in the presence of excess water (Biliaderis et al., 1980; Haase and Shi, 1991). For most starches this peak lies between 60 °C and 80 °C (Copeland et al., 2009). The second peak, around 90 °C, was credited to the denaturation of vicilin, while the third peak was credited to the denaturation of legumin at around 106 °C (Arntfield et al., 1985). DSC measurements of starch can also give rise to another peak (*M1*), especially at low moisture content, which represents the melting of the starch. This peak was not identified in this research.

#### 4.2.2 Starch gelatinization

For the samples analyzed, the *G* peak (the excess water gelatinization peak) occurred at 73.4 °C (±2.4 °C) (data not shown). The peak temperature did not correlate with the overall moisture content or the moisture content of starch in the dough (Equation (13)) ($R_{\mathrm{adj}}^{2}$ < 0.1), as is expected from literature (see Section 1). van der Sman and Meinders (2011) modelled the initial gelatinization of starch using free volume extension of Flory-Huggins theory and constructed a state diagram for starch, which shows limited influence of the mass fraction of water (*y*_*w*_) (which is equivalent to *mc*_*s*_(*dough*)) on the gelatinization temperature for *y*_*w*_ > 0.3 g g^−1^. They do, however, show a linear relation of the melting temperature of starch to the mass fraction of water, which describes the peak temperature of the *M1* peak. In this paper, the *M1* peak could not be identified, as its position (T) and size (Δ*H*) depend on the moisture content and it can overlap with the denaturation peak of vicilin and potentially also legumin. The possible effects of this on the interpretation of the denaturation temperature of legumin (*T*_*d*_) are discussed towards the end of Section 4.2.3.

**Fig. 5:**
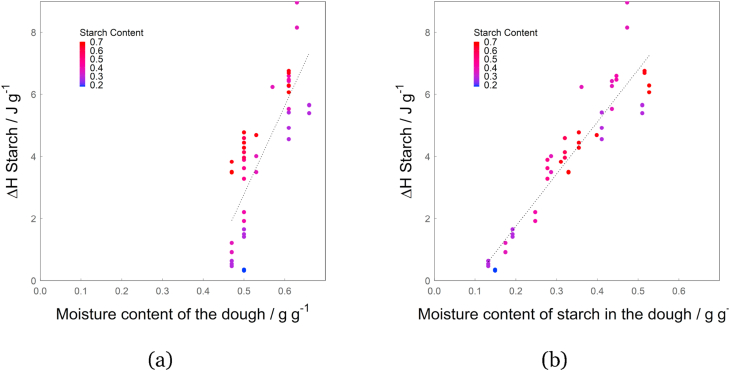
The enthalpy of the initial starch gelatinization (Δ*H*_*Starch*_) over (a) the overall moisture content of the dough and (b) the modelled moisture content of starch in the dough (*mc*_*s*_(*dough*)). Dotted lines are linear fits of Δ*H*_*Starch*_ with (a) *mc* and (b) *mc*_*s*_(*dough*) as variables, resulting in $R_{\mathrm{adj}}^{2}$ = 0.63 and $R_{\mathrm{adj}}^{2}$ = 0.87, respectively.

In Fig. 5, the Δ*H* over the overall moisture content of the dough is shown. There is no clear correlation of the two, resulting in an $R_{\mathrm{adj}}^{2}$ of the linear regression of 0.63. It is quite obvious, however, that Δ*H* depends on the starch content as well, since the high starch content samples (red) are underestimated by the linear regression, while the low starch content samples are mostly overestimated (purple, blue). Therefore, in Fig. 5b, Δ*H* is shown over *mc*_*s*_(*dough*), which is derived from Equation (13) which in turn depends on the overall moisture content and the starch content of the sample (Equation (11)). The use of a linear regression to correlate Δ*H*_*Starch*_ and *mc*_*s*_(*dough*) yields an $R_{\mathrm{adj}}^{2}$ of 0.87. This underlines that Equations (12), (13)) give a reasonable approximation of the distribution of moisture between the protein and starch phase. It is important to note that at high *mc*_*s*_(*dough*) starch always fully gelatinizes during the initial gelatinization and therefore Δ*H* becomes constant (Baks et al., 2008). This most likely occurs outside the regime of *mc*_*s*_(*dough*) investigated here, as (Habeych et al., 2009) and (Baks et al., 2007) found a Δ*H* of 13.4 ± 0.6 J g^−1^ and 13.4 ± 2.0 J g^−1^ for wheat starch at a moisture content of 0.91 g g^−1^ and 0.9 g g^−1^ respectively. The extrapolation of the current data set to *mc*_*s*_(*dough*) = 0.9 g g^−1^ yields a Δ*H* of 14.68 J g^−1^, indicating the validity of our data and the linear correlation of *mc*_*s*_(*dough*) and Δ*H*. For samples with low moisture content of starch in the dough, the starch does not fully gelatinize at the temperature of the *G* peak but undergoes further transition at higher temperatures. The degree to which starch gelatinizes at this temperature depends on the initial water content of the starch (Tananuwong and Reid, 2004), or in this case *mc*_*s*_(*dough*). At *mc*_*s*_(*dough*) below 0.3 g g^−1^, no gelatinization occurred at the initial gelatinization temperature.

This also suggests that the often described swelling of starch granules before or during initial gelatinization (Ai and Jane, 2015; Palanisamy et al., 2020) does not affect the moisture content of the protein phase or starch phase. Since the moisture content of the starch phase remains the same during swelling, the swelling of starch granules is caused by a migration of water within the starch phase. It is therefore likely that the moisture content of the starch phase consists of an extra-granular and an intra-granular water population, changing to only an intra-granular water population through swelling (Tananuwong and Reid, 2004).

#### 4.2.3 Protein denaturation temperature of mixed starch-protein gels

As mentioned in Section 4.2.1, three peaks were found in the thermograms depending on starch and moisture content. The second and third peak at around 90 °C and 106 °C respectively represent vicilin and legumin (Arntfield et al., 1985). As the third peak was the most distinct and with good resolution, it was chosen to represent the denaturation of protein. Besides, the effect of moisture content on denaturation temperature (*T*_*d*_) seems to be comparable for vicilin and legumin in the studied range (Arntfield et al., 1985), and the composition of the protein in the protein phase is unlikely to change.

**Fig. 6:**
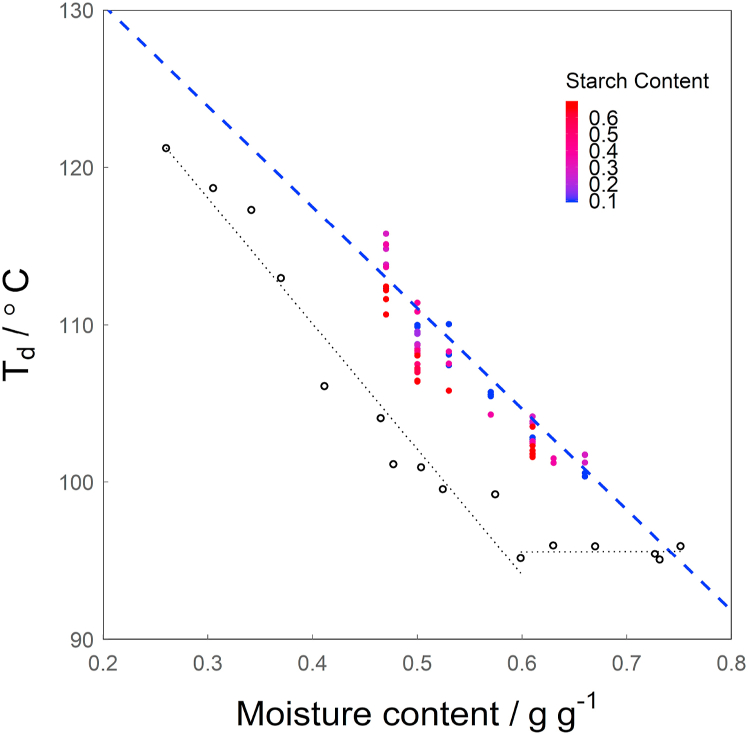
*T*_*d*_ measured using DSC over the overall moisture content of the sample (wb.). The open black symbols show data for legumin in faba bean protein isolate, taken from Arntfield et al. (1985). The blue dashed line is *T*_*d*_ for sc = 0, according to Equation (14), the black dashed lines are linear regressions of the data reproduced from (Arntfield et al., 1985) in the range where *T*_*d*_ depends on *mc* and where *T*_*d*_ remains constant irrespective of mc. (For interpretation of the references to colour in this figure legend, the reader is referred to the Web version of this article.)

Fig. 6 shows the denaturation temperature of legumin (*T*_*d*_) against the overall moisture content of the sample. By changing the starch content, the denaturation temperature of legumin decreased by maximally 5.12 °C, when using blends with an overall moisture content of 0.47 g g^−1^.

The potential influence of the starch content on the correlation between the moisture content and *T*_*d*_ can be incorporated in a model via Equations (7)–(10). Equation (9) was found to give the best fit based on the highest $R_{\mathrm{adj}}^{2}$, while the p-value was below 0.001 for all variables used in this model, showing that they are significant. Equation (10) resulted in the same $R_{\mathrm{adj}}^{2}$, but the p-values show that not all variables are significant. Summary tables of all fitted models can be found in the Supplementary information (Tables S5–S8). Fitting resulted in the following equation describing the relation between moisture content, starch content and the denaturation temperature of legumin in starch-protein gels:

(14) $$T_{d}=-64.16*mc-3.40*sc+143.15$$

where *T*_*d*_ is the denaturation temperature of legumin in °C, *mc* is the overall moisture content of the sample in g g^−1^ (wb.) and *sc* is the starch content in g g^−1^ (db.), resulting in an $R_{\mathrm{adj}}^{2}$ = 0.88.

Similarly to the approach used to determine the *mc*_*p*_(*dough*), setting *sc* to 0 after solving for *mc* yields the moisture content of the protein phase in the gel (*mc*_*p*_(*gel*)):

(15) $$mc_{p}\left(gel\right)=\frac{T_{d}-143.15}{-64.16}$$

where *mc*_*p*_(*gel*) is the moisture content of the protein phase in the gel in g g^−1^ (wb.) and *T*_*d*_ is the denaturation temperature of legumin in °C. This relation is shown in Fig. 6 by the dashed blue line. Furthermore, the values of denaturation temperature of legumin found by (Arntfield et al., 1985) using DSC are shown as well (open symbols). The relation of the moisture content of faba bean protein and these values can be split in two regimes: a linear decrease of *T*_*d*_ with increasing moisture content until 0.6 g g^−1^ and a constant *T*_*d*_ above 0.6 g g^−1^. Here we found a similar dependency of the denaturation temperature on moisture content in the protein phase as reported by Arntfield et al. (1985). They do, however, differ from our results in temperature, which could be due to possible differences in origin of the raw materials and the purification process used by them. In our paper, a less refined material is used, leaving the possible influence of other components like sugars, fibres and salts on the denaturation temperature. The purification of the faba bean protein used by Arntfield et al. (1985) could have partially denatured the protein, causing the shift of the curve to lower temperatures observed here. Most likely, the *T*_*d*_ of mildly refined faba bean fractions also does not decrease for moisture contents above the maximum moisture content used in this study.

**Fig. 7:**
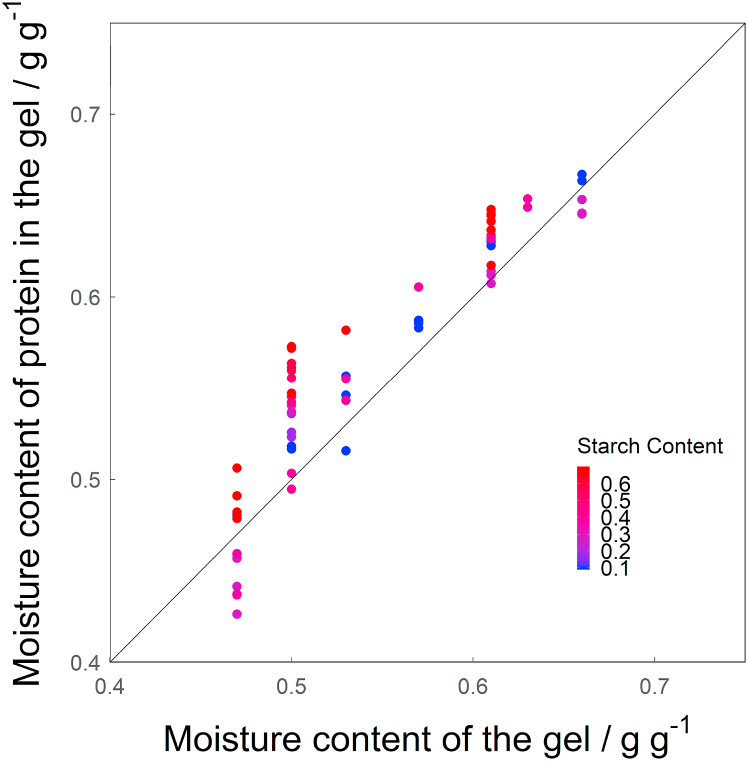
Modelled moisture content in the protein phase in the gel (*mc*_*p*_(*gel*)) over the moisture content of the gel. The solid line represents where the moisture content of the gel and the moisture content of the protein phase in the gel would be the same.

In Fig. 7, the modelled moisture content in the protein phase at denaturation is shown over the overall moisture content for all starch contents used (db.). Moisture content in protein at denaturation increases with starch content. At overall moisture contents >0.6 g g^−1^ the trend becomes less evident. This either means that the influence of starch on the moisture content of protein at denaturation is limited at high moisture contents (as is the case at room temperature), or that the influence of moisture content in protein on denaturation temperature becomes smaller at overall moisture contents >0.6 g g^−1^. The latter was also found to be true by Arntfield et al. (1985), who showed that the decrease of denaturation temperature of legumin with increasing moisture content levels off at 1.5 g g^−1^ (db.), which is equal to 0.6 g g^−1^ (wb.).

It is important to note that the starch melting peak (*M1* peak) was not identified in this research. It potentially overlaps with the *T*_*d*_ peak and interferes with the analysis. Hoseney et al. (1986) and Tananuwong and Reid (2004) show that the *M1* peak is rather broad and not a sharp peak like the *G* peak or the peaks for protein denaturation observed in this study. Such overlap is most likely at the lowest overall moisture content and starch content, as these conditions lead to the lowest values of *mc*_*s*_(*dough*)), which in turn lead to a high *M1* temperature. However, this also decreases the overall enthalpy of the starch gelatinization (since there is less starch in the sample), thereby decreasing the influence of the *M1* peak on the analysis of the denaturation peak of legumin and likely making this overlap less relevant. There is, however, the possibility of relevant overlap of the legumin peak and the *M1* peak at low moisture contents (0.47 g g^−1^) and intermediate starch contents (purple points), as these conditions lead to a relatively low *mc*_*s*_(*dough*), causing the *M1* peak to increase in height and appear at higher temperatures, and also lead to a smaller peak for the denaturation of legumin, as there is less protein present as the starch content increases. This could be the cause for the deviation of these samples from the rest of the data, observed in Figs. 6 and 7.

### 4.3 Comparing water distribution in doughs and gels

The comparison of the moisture content in the protein phase in the ingredients studied at room temperature (Fig. 3) with the moisture content in the protein phase at denaturation (Fig. 7) revealed that the latter is lower by up to 21.15%. The difference between them is larger for lower moisture contents and increases with starch content. Therefore, moisture had migrated from the protein phase to the starch phase during the heating process. A similar conclusion was drawn by Eliasson (1983) after studying the influence of wheat gluten on the gelatinization of wheat starch, using isolated ingredients. Since we have established that the moisture content of protein at room temperature is not influenced by the swelling of starch granules during their initial gelatinization (*G* peak), the redistribution of water must occur later, at higher temperatures. We suggest that this occurs during starch melting, which takes place at those higher temperatures. This is represented by the *M1* peak that most likely overlaps with the denaturation peaks of vicilin and legumin and can therefore not be detected. Melting of starch modifies the affinity of starch for water, creating a force “pulling” the water to the starch phase. Simultaneously, denaturation of protein exposes hydrophobic sites, creating a force “pushing” the water to the starch phase.

## 5 Conclusions

The distribution of water between the starch and protein phase in doughs and gels of mixed mildly-refined fractions of faba bean flour was determined in a moisture content range relevant for soft-solid foods such as plant-based meat and cheese alternatives. Quantification of the moisture content distribution was achieved by applying models based on established concepts such as sorption isotherms, mass balances and the moisture dependence of starch gelatinization and protein denaturation. The model based on sorption isotherms was used to determine the water distribution in a dough at room temperature, while the model based on the moisture dependence of protein denaturation determines the water distribution at elevated temperatures when protein denatures and gelatinizes. In the dough, protein takes up between 0.12 g g^−1^ and 0.28 g g^−1^ more water than starch. The amount of water associated with the protein phase in the gel is up to 0.12 g g^−1^ less than in the dough, showing the amount of water that migrated from the protein phase to the starch phase upon heating. The enthalpy change of the initial starch gelatinization correlates reasonably well with the calculated moisture content of the starch phase in the dough. Therefore, no or limited redistribution of moisture occurs between the starch and protein phase during the initial starch gelatinization, but at higher temperatures, during the melting of starch crystals.

This paper shows that it is possible to derive properties of components present in a flour or enriched fraction without having the components as pure ingredients. Such an approach will become more important when focusing on the use of enriched fractions rather than pure ingredients in future food applications. These findings have implications for the further development and design of production processes for aforementioned plant-based meat and cheese alternatives. The methods presented here can be used to predict and control ingredient properties that determine process parameters such as processing temperature. Furthermore, they underline the limitations of using overall moisture content without understanding water uptake and distribution in the individual components, all of which becomes even more important when using less refined ingredients.

## CRediT authorship contribution statement

**Jan M. Bühler:** Conceptualization, Data curation, Formal analysis, Methodology, Writing – original draft, Writing – review & editing. **Atze Jan van der Goot:** Conceptualization, Supervision, Writing – review & editing. **Marieke E. Bruins:** Conceptualization, Supervision, Writing – review & editing.

## Declaration of competing interest

The authors declare that they have no known competing financial interests or personal relationships that could have appeared to influence the work reported in this paper.
