# Supplementary material for: Quantifying water distribution between starch and protein in doughs and gels from mildly refined faba bean fractions
Source: Curr Res Food Sci. 2022 Apr 14;5:735–42. doi: 10.1016/j.crfs.2022.03.013 (PMC9046618; doi:10.1016/j.crfs.2022.03.013)
Supplement: Supplementary file 1 [file mmc1.zip › crfs_233_Supplementary information_V3_mmc1.odt]

Supplementary information

     Summary Tables for the models of the dough

adj

Table S1: Summary of model based on Equation 3. R2 = 0.00, R2

= 0.00

Term	Coefficient	Std. Error

intercept	0.52838	0.01275

sc	-0.0206	0.02954

t value

41.43

-0.697

Pr(>|t|)

<2e-16

0.487

adj

Table S2: Summary of model based on Equation 4. R2 = 0.66, R2

= 0.66

Term	Coefficient	Std. Error

intercept	-7.3749	0.4558

aw	8.0216	0.463

t value

-16.18

17.32

Pr(>|t|)

<2e-16

<2e-16

adj

Table S3: Summary of model based on Equation 5. R2 = 0.88, R2

= 0.88

Term	Coefficient	Std. Error

intercept	-9.53081	0.30446

aw	10.2824	0.31118

sc	-0.1896	0.01155

t value

-31.3

33.04

-16.41

Pr(>|t|)

<2e-16

<2e-16

<2e-16

adj

Table S4: Summary of model based on Equation 6. R2 = 0.90, R2

= 0.89

Term	Coefficient	Std. Error

intercept	-7.7394	0.4491

aw	8.4609	0.4576

sc	-6.5802	1.2474

aw:sc	6.4814	1.265

t value

-17.231

18.488

-5.275

5.123

Pr(>|t|)

<2e-16

<2e-16 4.52E-07

9.03E-07

     Summary Tables for the models of the gel

adj

Table S5: Summary of model for Td based on Equation 7. R2 = 0.00, R2

= 0.00

Term	Coefficient	Std. Error

intercept	107.2473	1.1068

sc	-0.7564	2.6465

t value

96.897

-0.286

Pr(>|t|)

<2e-16

0.776

adj

Table S6: Summary of model for Td based on Equation 8. R2 = 0.86, R2

= 0.85

Term	Coefficient	Std. Error

intercept	141.1	1.795

mc	-62.665	3.274

t value

78.61

-19.14

Pr(>|t|)

<2e-16

<2e-16

adj

Table S7: Summary of model for Td based on Equation 9. R2 = 0.88, R2

= 0.88

Term	Coefficient	Std. Error

intercept	143.1475	1.7248

mc	-64.1607	3.0064

sc	-3.3958	0.9188

t value

82.995

-21.341

-3.696

Pr(>|t|)

<2e-16

<2e-16 0.000476

adj

Table S8: Summary of model for Td based on Equation 10. R2 = 0.88, R2

= 0.88

Term	Coefficient	Std. Error

intercept	144.445	3.372

mc	-66.541	6.107

sc	-7.073	8.248

mc:sc	6.81	15.177

t value

42.835

-10.895

-0.858

0.449

Pr(>|t|)

<2e-16

9.40E-16

0.395

0.655
